# Membranes with the Same Ion Channel Populations but Different Excitabilities

**DOI:** 10.1371/journal.pone.0034636

**Published:** 2012-04-16

**Authors:** Marco Arieli Herrera-Valdez

**Affiliations:** 1 Department of Mathematics, University of Arizona, Tucson, Arizona, United States of America; 2 Evelyn F. McKnight Brain Institute, University of Arizona, Tucson, Arizona, United States of America; 3 Department of Mathematics and Physics, University of Puerto Rico in Cayey, Cayey, Puerto Rico; 4 Institute for Interdisciplinary Research, University of Puerto Rico in Cayey, Cayey, Puerto Rico; Georgia State University, United States of America

## Abstract

Electrical signaling allows communication within and between different tissues and is necessary for the survival of multicellular organisms. The ionic transport that underlies transmembrane currents in cells is mediated by transporters and channels. Fast ionic transport through channels is typically modeled with a conductance-based formulation that describes current in terms of electrical drift without diffusion. In contrast, currents written in terms of drift and diffusion are not as widely used in the literature in spite of being more realistic and capable of displaying experimentally observable phenomena that conductance-based models cannot reproduce (e.g. rectification). The two formulations are mathematically related: conductance-based currents are linear approximations of drift-diffusion currents. However, conductance-based models of membrane potential are not first-order approximations of drift-diffusion models. Bifurcation analysis and numerical simulations show that the two approaches predict *qualitatively* and *quantitatively* different behaviors in the dynamics of membrane potential. For instance, two neuronal membrane models with identical populations of ion channels, one written with conductance-based currents, the other with drift-diffusion currents, undergo transitions into and out of repetitive oscillations through different mechanisms and for different levels of stimulation. These differences in excitability are observed in response to excitatory synaptic input, and across different levels of ion channel expression. In general, the electrophysiological profiles of membranes modeled with drift-diffusion and conductance-based models having identical ion channel populations are different, potentially causing the input-output and computational properties of networks constructed with these models to be different as well. The drift-diffusion formulation is thus proposed as a theoretical improvement over conductance-based models that may lead to more accurate predictions and interpretations of experimental data at the single cell and network levels.

## Introduction

Electrical signaling allows fast transfer of information within and between cells. Electrical signals are produced by ionic transport within tissues, and in particular, across the membranes of cells. Most transmembrane ionic transport is mediated by membrane-spanning proteins that may either mechanically translocate ions across the membrane (transporters), or facilitate ionic diffusion by forming pores [1]. The dynamics of membrane potential can be modeled from a macroscopic perspective by assuming that the membrane, its channels and transporters, and the permeable ions on both sides of the membrane are equivalent to an electrical circuit [2], [3]. In this description, the total current through the membrane is the sum of the currents mediated by channels and transporters. Currents mediated by ion channels are typically modeled as the product of a conductance and a linear function of membrane potential [4]–[7]. This approach will be referred to herein as *conductance-based* (CB). However, ionic transport through channels is driven by electrical drift, as assumed in CB models, but also by diffusion [8], [9] which is not included in CB formulations.

**Figure 1:**
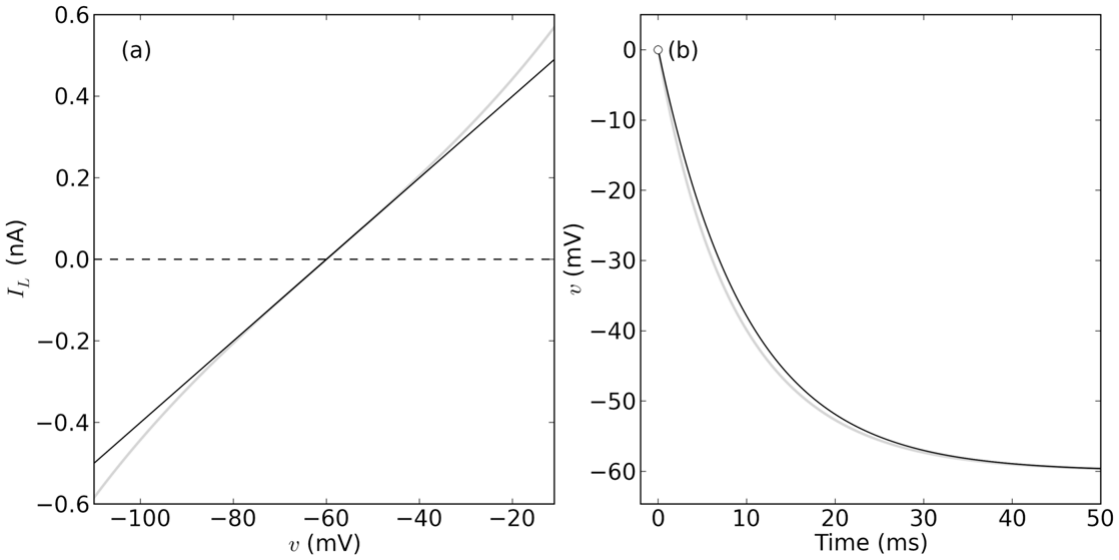
DD and CB currents and convergence to steady state. (**a**) DD and CB currents in gray ( Eq. 1 ) and black ( Eq. 2 ) respectively. (**b**) Convergence to steady state in a model of membrane potential ( Eq. 14 ) with dynamics as in Eqs. 15–16.

Expressions for transmembrane ionic flux that take diffusion into account can be derived using the Nernst- Planck equation [10], and used to describe transmembrane currents as already done by Goldman [11] and others [12]–[15]. These currents will be herein called *drift-diffusion* (DD) and models of membrane potential constructed using CB, or alternatively, using DD currents will be referred to as CB or DD models. CB models are generally regarded as good descriptions of membrane potential, have been studied extensively [16]–[20], and are thus very popular and used along with experiments to study cellular excitability [2], [3], [21]–[25]. On the other hand, DD models are more realistic [11], [26] but are not widely used in the literature. For instance, DD currents capture important nonlinear phenomena like rectification; a property that CB models cannot reproduce. In fact, as shown in the following paragraphs, the CB formulation for current is a *linear approximation* of its DD counterpart around the reversal potential of the current ( Fig. 1 a). DD and CB models reproduce basic features of the behavior of excitable cells [13], [19]. However, the nonlinearities contributed by DD formulations may result in very different dynamics in comparison to CB models. It is therefore important to ask to what extent the two approaches lead to qualitatively and quantitatively similar behaviors, everything else being equal. In other words, are CB and DD formulations computationally equivalent? If so, to what extent?

Models of excitable cells are used to understand the role of ionic currents on cellular signaling, make testable quantitative predictions, and interpret experimental results. Therefore, it is crucial to understand more about how the dynamics of the membrane change with the DD or CB formulation. To start addressing this question, two low-dimensional versions of the same model of membrane potential, CB and DD respectively, are constructed. The two models are assumed to have identical ion channel populations mediating a leak current and two voltage-gated currents, namely, a transient sodium (Na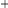) current and a delayed rectifier potassium (K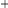) current [19], [27], [28]. The comparison is done by examining the bifurcation structure and behaviour of the two models in response to constant and time-dependent current stimulation, and synaptic input. In each case, different patterns of ion channel expression were taken into account. The qualitative features of the dynamics in the two models having identical ion channel populations are different, as predicted by their non-topologically equivalent phase spaces and bifurcation structures. For constant stimulation, the smallest sustained current amplitude that causes a transition between rest and repetitive oscillations in the membrane potential, or 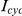 for short, is shown to differ in the two models. In a more dynamical setting, the *recruiting current*, defined as the smallest amplitude in an up-going ramp stimulus that results in action potentials, is shown to be significantly smaller than 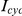 for both models, and smaller in the DD model than in the CB model. The excitatory synaptic current that causes repetitive spiking is also shown to be smaller than 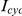, and repetitive spiking in response to synaptic input requires a smaller number of synapses in the DD model in comparison to the CB model. In sum, repetitive spiking occurs for smaller stimulus currents, and within a smaller range in the DD model in comparison to the CB model. The results presented here can be modified and extended for the study of other excitable membranes.

## Methods

### Electrodiffusion currents and membrane potential

The formulation for transmembrane currents driven by drift and diffusion used here is a generalization of a derivation based on first principles of thermodynamics and electrochemistry previously reported in [13] and expanded in [29]. The derivation starts by considering the ionic flux through open pores across the membrane written as the sum of electrical drift and diffusion with the Nernst-Planck equation (see Text S1 and [9], [12]). In brief, the cross-sectional area of the pore region inside an ion channel and the electric field across the membrane are assumed to be smoothly varying functions of distance along the pore. Assuming the flow of charge is stationary and integrating the equation between the intra- and extra-cellular domains along the pore axis allows writing an expression for the transmembrane current as a function of the membrane potential 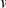. As a result, the current carried by ions of type 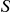 as they electrodiffuse through an open pore can be written as
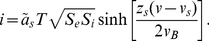
(1)where 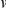 is the membrane potential, 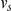, 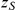, 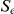, and 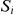 are the Nernst potential, valence, extracellular and intracellular concentrations of the ion 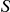 [13]. The term 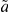 is a constant approximation to a function that depends on the properties of the pore, the electric field across the membrane, the mobility of 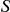, and other factors [30]. The Goldman constant field approximation [11] can be obtained as a particular case of Eq. 1 if it is assumed that the electric potential inside a channel is a linear function of the distance along the channel pore, and that the pore has constant cross-sectional area [13], [27]. The potential 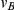 is the quotient 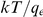 where 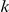 is Boltzmann's constant, 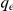 is the elementary charge, and 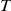 the absolute temperature. The current in Eq. 1 can be regarded as a macroscopic description of the transmembrane current produced by ions of type 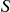 as they diffuse through an open channel and will be herein referred to as a DD current. For contrast, the CB current through an open channel permeable to 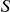 is a linear function of 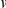 (Fig. 1 a) given by
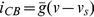
(2)where 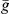 represents the maximal conductance of the current carried by 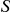-ions. For K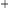 and Na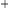 ions, but not for Ca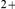, the concentrations 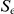 and 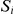 can be regarded as constants [4], [27].

### Whole-membrane currents

The formulation in Eq. 1 can be extended to consider the current mediated by several hundreds or thousands of gated channels. Let channel gating be represented by a number 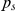 between 0 and 1 that depends on the gating mechanism of the channel. The *gated* whole-membrane current can be written as:
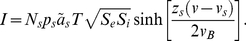
(3)where 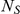 is the number of channels in the membrane. Loosely speaking, the quantity 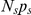 can be thought of as the average number of open channels permeable to 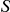 (see [31] for an interesting perspective in this regard). If the absolute temperature and the transmembrane concentrations of Na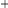 and K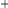 are assumed to be constant [4], [27], then
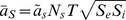
(4)can be thought of as a constant representing the maximum current through the membrane.

Voltage-dependent gating is described from a macroscopic perspective as a first-order process with steady states written explicitly in terms of the change in free energy caused by the conformational changes that underlie channel gating. Ligand-gated channels are modeled following previous work by Destexhe *et al.* [32].

### Membrane potential

The comparison between the DD and CB models is done with a two-dimensional dynamical system defined in terms of a *core* set of currents: a leak current, two voltage-gated currents carried by Na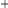 and K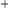, and a stimulus current representing either stimulation through an electrode, or fast excitatory synaptic input. Importantly, all the currents are written using the same gating functions and therefore, the only differences between the two models are in the driving force portion of the currents. An implicit assumption in this construction is that additional membrane channels and transporters fulfill a complementary, but not necessary, role in producing rest-to-spiking transitions, as explicitly illustrated, for instance in [27].

The membrane potential is represented by the variable 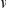 with dynamics defined by:
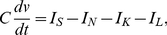
(5)

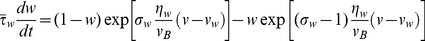
(6)where 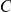 is the membrane capacitance and 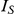, 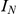, 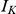, and 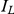 represent, respectively, current from an external stimulus, voltage-gated Na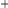 and K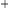 currents, and a non-gated leak current (see Table 1). The variable 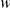 represents the dynamics of the K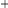 channel activation. The gating charge and half-activation potential of 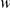 are, respectively, 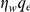 and 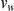. The basal rate of the gating reaction, 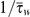, is a function of temperature [7]. Since all the simulations presented here are assumed to occur at 22
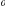
C, 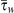 becomes a constant. The peak and symmetry of the time constant as functions of 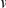 are controlled by 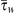 and 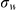, respectively [29], [33], [34]. Na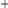 channel inactivation and K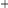 channel activation are linearly coupled [18], [19], [27]. Steady state activation for Na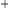 channels is given by 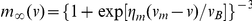. Parameters for the simulations can be found in Table 2.

**Table 1:** Functional forms of the different transmembrane currents.

Current	DD	CB
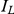	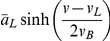	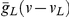
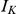	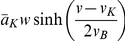	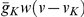
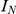	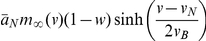	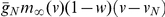
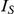	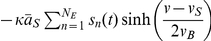	− 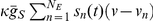

Gating is the same in both formulations.

**Table 2:** Parameters and Constants.

Name	Value	Units	Description
Physical constants			
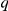	1.60217733 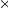 10 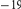	C	Elementary charge
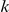	1.3806582 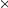 10 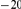	mJ/K	Boltzmann's constant
Membrane properties			
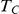	22	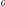 C	Room Temperature 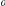 C
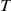	273.15+ 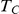	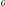 K	Absolute temperature
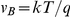	25.43	mV	Boltzmann's potential
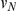	70	mV	Reversal potential for Na 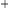
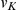	−90	mV	Reversal potential for K 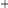
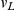	−60	mV	Reversal potential for membrane leak 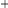
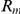	100.0	M 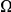	Membrane resistance
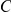	0.1	nF	Membrane capacitance in adult *Drosophila* MN5 [84]
Channel kinetics			
Transient Na from Adult *Drosophila* DmNav1 [42]			
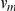	−29	mV	Half-activation
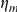	2.0		Gating charge of activation
K-delayed rectifier from *Drosophila* Shab [38]			
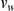	−1	mV	Half-activation
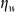	2.0		Gating charge of activation
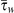	10	ms	Max. activation time constant
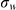	0.6	-	Symmetry of activation time constant
Maximum current amplitudes and conductances			
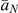	10.0	nA	NaT maximum current amplitude
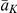	25	nA	Kd maximum current amplitude
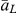	0.5	nA	Leak maximum current amplitude
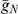	0.2	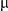 S	NaT maximum conductance
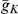	0.5	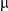 S	Kd maximum conductance
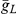	0.01	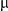 S	Leak maximum conductance
Normalized model			
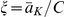	100	nA/nF	20.4 Scaling factor for physiologically relevant 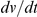
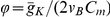	1.96	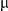 S/nF	
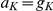	2.5	nA	Maximal current amplitude for potassium relative to 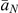 .
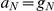	1	nA	Normalized maximal current amplitude for Kd
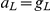	0.05	nA	Normalized maximal current amplitude for Kd

The *stimulus current* is either a constant (used as a bifurcation parameter), a time-dependent function representing external stimulation, or a time- and voltage-dependent function representing synaptic input. The smallest 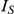 necessary to cause a transition between rest and sustained oscillations with a square pulse will be referred to as 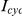. The *time-dependent stimulation* will consist of 5 epochs: (1) bottom, with stimulus amplitude 0 nA, (2) ramp up (*Up*), (3) constant stimulation with amplitude equal to the maximum reached by the ramp (*Top*), (4) ramp down (*Down*), and (5) bottom again. This stimulation protocol will be referred to as up-top-down (UTD). Unless otherwise specified, *Up*, *Top*, and *Down* epochs have the same duration with 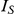 being continuous as a function of time. For 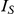 with a ramping stimulation, the minimum current amplitude required to start sustained oscillations during *Up* or *Top* will be called *recruitment* current, and the stimulus amplitude during *Down* at which a transition between sustained oscillations and rest occurs will be called *de-recruitment* current.

*Synaptic input.* The activity of the presynaptic cells is simulated by generating 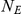 independent *spike trains* with gamma-distributed interspike intervals, each with a mean rate 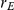 [34], [35]. For simplification purposes, it is assumed that an action potential in each of the input neurons activates, on average, 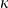 synapses after a presynaptic action potential [35], [36], each synapse having a maximum postsynaptic current amplitude 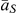. Each synapse made by the 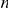
th presynaptic cell is gated with a time-dependent probability of opening 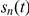 with dynamics defined by
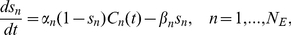
(7)where 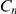 is the concentration of neurotransmitter for each of the 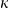 synapses activated by the 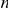
th presynaptic neuron at times 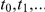 (see [32] and Fig. S2 and Table 3). The time-course of neurotransmitter concentration in the cleft is given by
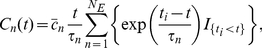
(8)where 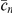 represents the maximum neurotransmitter concentration at synapses from the 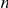
th presynaptic contact. The indicator function 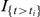 is equal to 1 if 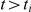 and zero otherwise [5], [7].

**Table 3:** Parameters for simulations with time-dependent stimulation or synaptic input.

Name	Value	Units	Description
nAChRs from adult *Drosophila* central synapses [44]			
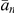	10	nA	Maximum mEPSC amplitude
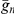	0.2	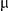 S	Maximum conductance of mEPSCs
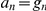	0.4	-	Normalized mEPSC amplitude relative to 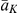
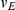	0	mV	Reversal potential
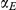	1	mM/ms	Forward rate of postsynaptic activation
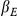	0.2	1/ms	Backward rate of postsynaptic activation
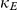	10	-	Average number of activated synapses per spike
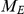	[0,10 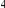 ]		Number of excitatory synaptic axons
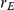	7	Hz	Input rate of each excitatory axon

### Reparametrization in terms of relative channel contributions

Recall that the CB approximation to the DD current is valid only for voltages near the reversal potential of the current. Therefore, for any given potential, the DD model (Eq. 5) cannot be written as a CB model by simply replacing the DD currents by their CB approximations. However, it is still possible to investigate whether the dynamics from the DD model are qualitatively different to those of the CB model by rewriting Eq. 5 so that the relative contributions of the channels to the change in membrane potential are the same. To do so, start by multiplying the right hand side of the DD model by a normalization current 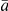, and divide each of the currents by 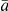; do the same for the CB model using a normalization conductance 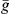. As a consequence,
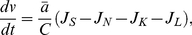
(9)with 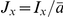, for 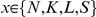. The remaining coefficients leading each of the currents can then be set to be equal in both versions of the model:
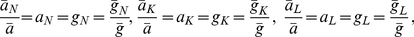
(10)with 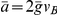. If 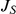 represents synaptic input, the amplitudes for the currents corresponding to each of the input axons can also be set as
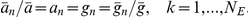



The normalization term for the DD model could be 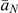, 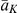, 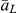, 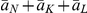, or any other convenient current; the same applies for 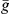 in CB models. The choice 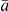 allows, however, different interpretations for the model. For instance, 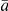 could be the sum of all the amplitudes for DD and similarly for 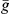, in which case the coefficients in front of each current can be thought of as weighted by a maximum total current (or conductance). Recall that the maximum amplitude of each of the DD currents (alternatively, maximum conductance for CB) can be thought of as multiple of the number of channels mediating the current, so these normalizations allow interpretations in terms of *relative* expression of ion channels. For instance, if all the currents in the DD model are divided by the maximum amplitude of the Na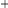 channel (and their CB counterparts are divided by 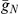), then 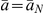 and the ratios 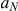, 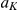, and 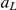 in the right hand side of Eq. 9 can be thought of as amplitudes relative to the number of Na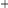 channels in the membrane. Then, as indicated by Eq. 4,
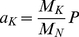
(11)where 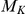 and 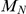 are the numbers of K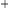 and Na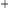 channels and 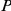 is a constant. Therefore, 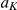 is proportional to the ratio of K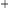 to Na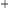 channels. In other words, 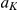 can be thought of as an indicator of the *relative expression* of K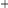 delayed rectifier channels in the membrane with respect to the expression of Na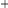 channels.

### Parameters and fits to experimental data

The channel kinetics used here are based on the biophysical properties of delayed-rectifier K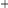 channels expressed in somato-dendritic compartments encoded by the *Shab* gene in central neurons of adult *Drosophila* [37]–[39], or one of its vertebrate homologs (*e.g.* Kv2.1 [40]). The Na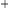 channels can be thought of as one of the protein products of the *para/DmNav* gene also present in adult *Drosophila* [41], [42] or one of its vertebrate homologs (*e.g.* Nav1.1–1.9 in vertebrates [43]). Synaptic currents are assumed to be excitatory and mediated by fast cholinergic receptors, one of the main mechanisms of excitation in invertebrate central synapses [44] also present in the central, peripheral, and enteric nervous systems of vertebrates [45]–[47]. In all the simulations presented here, each of the input axons is assumed to have an average firing rate of 7 Hz with gamma-distributed interspike intervals. Each action potential from an input cell is assumed to activate (on average) 10 synapses from its collateral terminals, each producing an excitatory post-synaptic current [35]. Data for the channel parameters was obtained by fitting digitized current traces recorded under voltage clamp mode and reported in [39] and [42]. Digitalization was done with custom code (Fig. S1 and Table 2). Once the parameters for the channels are fixed, the only free parameters left in both CB and DD models are the maximum amplitudes and conductances, respectively. The differential contribution of the channels to the excitability of the membrane can then be directly assessed by considering the ratios of the maximum amplitudes (DD) or conductances (CB) of the different currents in the model. The parameters 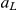 can be determined by using the input resistance as illustrated in later paragraphs. 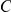 and the maximum 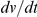 are found directly from recordings.

### Initial choices of parameters

The normalizing amplitude 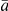 can be found using 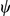 and 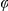, with 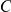 to fit the maximum rate of change in the model to the one obtained in recordings. This means one more parameter can be fixed if 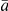 is either 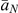 or 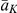. A particularly convenient choice used here is 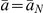, because the magnitude of 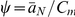 matches the magnitude of the desired maximum 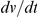. The combined parameters 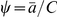 and 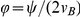 from Eq. 9 can then be used to constrain the model to represent different cell types because they control the maximum 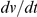, which can be determined from recordings. As a rule of thumb, the maximum 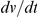 should be less than 50 mV/ms for cardiac myocytes [48]–[50] and pancreatic beta cells [51]. In neurons, the maximum 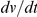 may reach 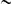
300 mV/ms (see for instance, [52]). For instance, for the model presented here, 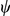 can be set to about 100 nA/nF (with 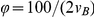). If the membrane capacitance is 0.1, then 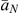 = 10 nA. A starting value of 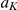 = 3 (as rule of thumb between 2 and 10) leaves 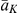 = 30 nA. Notice that if 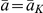, the rule of thumb can be applied by algebraically rearranging the terms, which yields 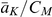 = 300 nA/nF (because 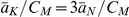) and 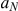 = 0.33.

The comparisons between DD and CB models shown subsequently are made assuming 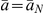 with maximum potassium current amplitudes between 1 and 5 times larger than the maximal amplitude of the sodium current, corresponding to relative level of expression in the K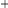 channels 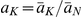 within the interval 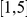. The range was determined by a global exploration of the bifurcation structure of the models in codimension 1 using 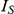 as the bifurcation parameter. The analysis of the model will be focused on the transitions into and out of repetitive spiking as dictated by varying the relative contributions of the different currents to the change in membrane potential.

### Bifurcation analysis and associated membrane potential behaviors

The *steady state currents* are obtained after excluding 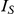 from Eq. 5 and replacing 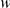 by its steady state 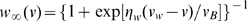 in the voltage-gated currents. The resulting curve, called 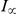 herein, is used to calculate the fixed points 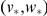 of the system. If 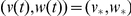, then neither 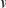, nor 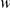 change. Trajectories that pass through focus points are *spirals*, which means the membrane potential oscillates when the system is near a focus point. In contrast, the membrane potential does not oscillate when the 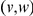 is near nodes. Trajectories near *saddle node* points initially move toward the saddle-node and eventually diverge from it, which means that if 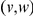 is near a saddle-node, 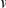 will eventually move away from the 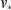-value of the saddle-node. The *cycles* of the system represent sustained oscillations in the membrane potential. *Limit cycles* are asymptotically stable attractors. This means that the membrane potential will go into sustained oscillations if the 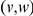 is within the basin of attraction of a limit cycle. Sustained oscillations are regarded as repetitive spiking if their amplitude is 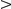
30 mV and their maximum 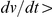
10 V/s. The system is *bistable* if it has two attractors (e.g. a fixed point and a limit cycle).

The system in Eqs. 5–6 has at least one asymptotically stable attractor for parameters within the physiologically meaningful range. In other words, there should be either a fixed point or a limit cycle (sustained oscillation) that the system goes back to. If all the fixed points are unstable, there is no resting membrane potential and sustained oscillations are expected to occur (a limit cycle is expected to exist [6], [7], [53]). The 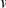-value of an asymptotically stable fixed point can be regarded as a *resting potential* (especially if near −60 mV). Asymptotically stable *focus* points are such that the membrane potential oscillates toward the resting value. In contrast, the membrane potential converges to asymptotically stable *node* points monotonically (without oscillating). Recall that a *bifurcation* occurs when either the number, the type, or the stability of the fixed points or cycles of the system change [54]. That is, a bifurcation indicates a qualitative change in the behavior of the system; for instance, a transition between rest and sustained spiking. Note therefore, that the analysis presented here links patterns of relative ion channel expression with bifurcation structure.

### Bifurcations

The points shown in bifurcation diagrams are color-coded based on the characteristics of the eigenvalues of each fixed point. *Asymptotically stable* fixed points are represented with small solid dots. *Unstable* fixed points are represented with circles. 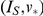 pairs corresponding to *focus* points are shown in black. The pairs 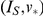 corresponding to *node* points are shown in blue. 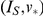 from *saddle-node* points are shown in green.

*Andronov-Hopf* (AH) bifurcations occur when a focus point changes in stability. Subcritical and supercritical AH bifurcations are associated to bistable and monostable systems, respectively. Systems like Eqs. 5–6 that undergo a subcritical AH bifurcation, typically loose or gain an unstable limit cycle. In the former case, the membrane is typically bistable, with a limit cycle and a stable fixed point separated by an unstable cycle. At the bifurcation the unstable cycle closes into the fixed point and dissappears, leaving an unstable focus and the limit cycle around it. Prior to the bifurcation the system could go to rest or into repetitive spiking depending on its initial conditions. After the bifurcation, the system is monostable and its only asymptotic behavior is the sustained oscillation (repetitive spiking). In *Saddle-node* (SN) bifurcations the number of fixed points changes between 3 and 1 (or viceversa) and are associated with non-monotonic 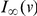 curve. Repetitive spiking may emerge through a SN bifurcation if, for example, the remaining point is unstable and a limit cycle remains as the only attractor of the system. A *fold limit-cycle* (FLC) bifurcation is such that two cycles appear or disappear (similar to SN). One cycle is unstable and surrounds the stable fixed point. The other cycle is stable and surrounds the unstable cycle. The unstable cycle delimits the basins of attraction of the fixed point and the limit cycle. FLC bifurcations occur near subcritical Andronov-Hopf bifurcations.

The values used in the simulations presented here can be found in Table 2. Deviations from the parameter set used in the tables are noted in the figures.

### Numerical solvers

Numerical simulations shown in this manuscript were performed using the solver integrate.odeint (lsoda from the FORTRAN library odepack) available from the Python module scipy (Python Software Foundation, http://www.python.org).

## Results

### Conductance-based currents are first-order local approximations of electrodiffusion currents

**Figure 2:**
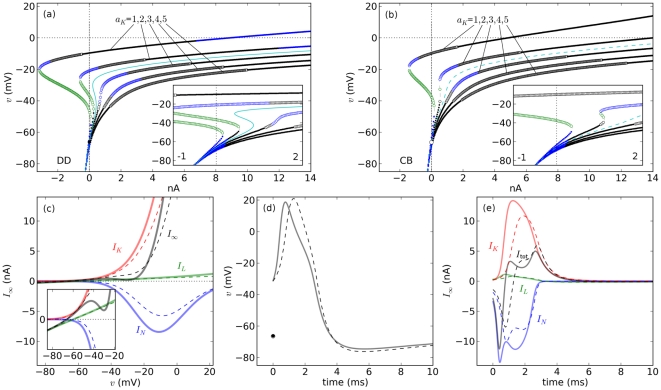
Bifurcation diagrams and trajectories. DD (solid) and CB (dashed). Panels (**a**) and (**b**) show the fixed points (by type) as a function 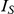, for relative expressions of K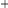 channels 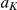 = 1,2,2.5,3,4,5. The fixed point curve for 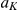 = 2.5 is shown in cyan. (**c,d,e**) Steady state currents, action potential, and its underlying currents, respectively, with 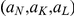 = (1,2.5,0.05). 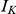, 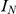, 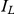, and 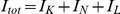 are shown, respectively, in red, blue, green, and black. The time course of an action potential after a 35 mV shift from rest and its underlying currents are shown in (**d**) and (**e**), respectively. The black dot and the surrounding circle mark the resting potentials for the DD and CB models are illustrated.

The CB and DD formulations are mathematically related. To see it, consider the Taylor series of the hyperbolic sine around 0 truncated to first-order: 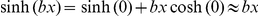. This means that Eq. 1 can be approximated around 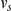 as follows:
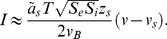
(12)The quotient in front of the voltage difference in Eq. 12 is a conductance (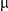
S), which can be rewritten as
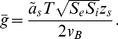
(13)The CB expression for current from Eq. 2 is thus a first-order approximation of the DD current near the reversal potential 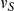
(see Fig. 1 (a) and green trace for 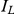 in Fig. 2 ). If the membrane input resistance (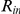) is known, the relationship between the maximum conductance and the maximum current in Eq. 13 allows calculation of 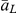. To do so, assume 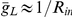 and calculate the amplitude of the leak current as 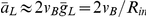 using the relationship between the DD and CB currents described in Eq. 13.

To obtain some intuition about the role of the leak current, and in particular 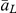 (or 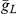), in shaping the asymptotic behavior of 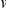, it is useful to consider a reduced version of Eq. 5 that only includes the leak current:
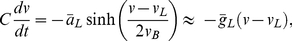
(14)where 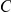 is the membrane capacitance ( Fig. 1 b). Assuming 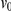 as an initial condition, the first equality in Eq. 14 (DD) gives a membrane potential of the form
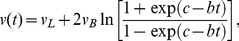
(15)where 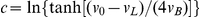 and 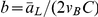. The solution corresponding to the CB approximation on the right of Eq. 14 is
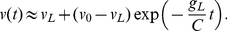
(16)It can be readily seen in both cases ( Fig. 1 b) that 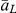 (or 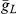) modulates the time constant that governs the return of 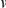 in Eq. 14 to its resting value, 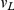. This explains why a larger leak current would lead to faster return to the resting potential. Larger values of 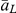 yield smaller membrane time constants, which results in faster convergence toward rest (see [55]–[58]). Note the convergence to 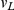 is slightly different for the two models.

### Different behaviors for the same ion channel expression

As noted earlier, direct substitution of the DD or CB formulations into Eq. 5 may result in different excitability profiles. These differences can be examined from a macroscopic perspective using *bifurcation analysis* for different choices of the relative contribution of the K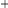 current with respect to Na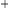, 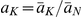 (with fixed 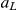). To do so, 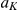 is fixed and the *fixed points* of the system and their types are found as a function of the external current 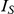 (Fig. 2 a–b). In general, as 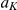 increases, the shape of the fixed point curve as a function of 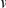 changes from non-monotonic to monotonic in both models, but monotonicity emerges in CB models for smaller 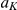. The two models display, however, important differences in regard to the *number* and type of their fixed points depending on the relative levels of ion channel expression.

If the relative contribution of K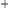 and Na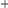 channels is the same (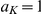, first curve from left to right in Fig. 2 a–b), both DD and CB models have three fixed points when 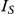 = 0. In the DD model, the two fixed points with lowest and highest 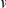-values correspond to asymptotically stable focus points, the remaining one corresponds to a saddle point. The system is thus bistable: the smallest 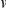-value corresponds to the resting membrane potential and the largest 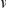-value corresponds to a depolarization block potential. In an experiment, this would mean that the membrane potential could be block-depolarized from its resting value by a brief but large enough pulse of current, or taken back to rest by down-shifting 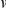 with a negative short pulse of current. The number of fixed points decreases from 3 to 1 for larger stimulus amplitudes (SN bifurcation); the fixed point that remains is stable. Experimentally, this is a case in which square pulses of current injection would depolarize the membrane, perhaps generating a pulse that would end at a depolarized membrane potential. In this case no stimulus would result in repetitive spiking (see for instance Fig. 1 in [59] and Fig. S2). In contrast, in the CB model has only one stable fixed point for 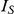 = 0. The only stable fixed point disappears in a SN bifurcation as 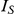 increases, leaving behind an unstable point and a limit cycle (not shown). As a consequence, the CB model predicts sustained spiking for large enough 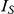 if 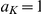.

If the maximum K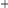 current amplitude is twice as large as the maximum Na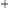 current amplitude (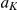 = 2), the DD model has three fixed points at 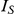 = 0 and the CB model has only one (second curve from left to right in Fig. 2 a–b and Fig. S3). In this case, the fixed point curves of the two models are non-monotonic as a function of 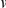. Importantly, the transition into repetitive spiking with square pulses of current will occur in this case near a saddle node bifurcation in the DD model, and through a FLC bifurcation in the CB model (see inset in Fig. 2 b inset, Fig. S4, and Text S1).

If the expression of potassium channels is higher, say 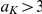 ( Fig. 2 a–b, lower 3 curves), then both models have only one fixed point for all 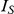 considered here (the fixed point curves are monotonic as functions of 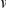). In all of these cases there are sustained oscillations that emerge through a FLC bifurcation (near Hopf points). These dynamics are like those observed in experiments where an up-going ramp current stimulus produces small subthreshold oscillations before repetitive spiking starts [60]. A similar phenomenon occurs if a large enough stimulus amplitudes, when repetitive spiking disappears with an oscillation toward a depolarization block.

The DD and CB models also exhibit *different sequences* of fixed point bifurcations as 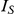 increases for each of the 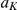
's under consideration. For instance, for 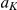 = 4 (second curve from right to left in Fig. 2 a–b), the sequence of fixed points in the DD model includes stable nodes (ca. 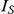 = 0), then stable foci that become unstable and later turn into unstable nodes, then turn into unstable foci again that become stable (ca. 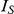 = 11), etc. In contrast, the CB model has stable nodes first (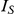 = 0), then stable foci that become unstable and then stable again without turning into nodes (ca. 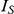 = 13), etc.

In sum, unstable nodes and foci appear earlier in the DD model (with respect toCB) as 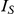 increases (Fig. 2 a–b) and the minimal current stimulus that evokes repetitive spiking, 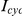, is smaller for the DD model in comparison to the CB model, for 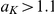. The *range* of 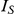 for which repetititive spiking occurs is also smaller for DD models than for CB models for any given 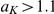. Experimentally, this means that the CB model requires larger depolarizations from rest in comparison with the DD model in order to show an action potential (see Figs. S4 and S5). In addition, the above observations highlight potentially different mechanisms underlying transitions into or out of repetitive spiking as a function of the relative expression of ion channels. Taken together, past paragraphs show that, in general, the dynamical systems that result from using DD and CB formulations in Eqs. 5–6 are *non-topologically equivalent* despite having identical populations of voltage-gated channels. In other words, the DD and CB models yield membranes with *different electrophysiological signatures* despite of having *identical channel expression*.

### Rest-to-spiking transitions

The choice of 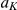 = 2.5 results in DD and CB models that are readily seen as not topologically equivalent because their 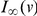 curves are non-monotonic and monotonic, respectively (cyan curves in Fig. 2 a–b, and Fig. 2 c–e). As a consequence, the dynamical behaviors of the two models are qualitatively different as well. For this reason, a more detailed comparison between DD and CB models is carried out from herein with 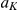 = 2.5.

First, note that gating causes the divergence between the steady state 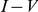 relationships of the CB and DD formulations to be more noticeable for K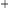 currents than for Na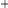 currents [14], [61]. To compare the dynamics in the two models, it is useful to consider the trajectory described by the system when an action potential occurs (Fig. 2 d). If the membrane potential is depolarized 35 mV from rest the action potentials in the two models are comparable in amplitude and duration, and they both go back to very similar resting potentials. However, the nonlinearities from the DD formulation can be observed in faster upstroke and initial downstroke, relative to the CB model. A closer inspection of the Na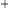 and K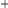 currents shows an earlier activation in the DD model relative to the CB model ( Fig. 2 e). This time delay in the activation of the voltage-gated currents is accentuated for larger values of 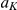, especially for the Na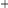 current (see Fig. S5).

### Different behaviors for the same stimulus current

**Figure 3:**
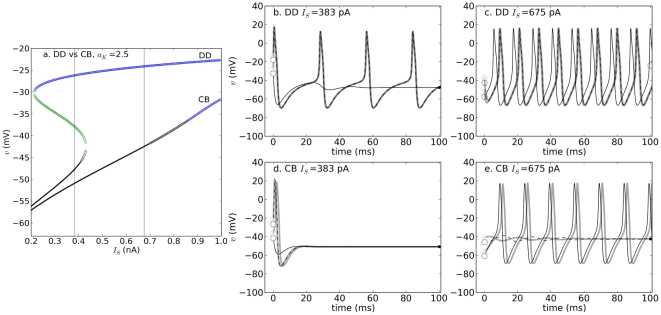
Bifurcation structure and dynamics of DD and CB models for 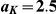. a. Bifurcation profiles. **b–e.** Dynamics of the DD (**b,c**) and CB model (**d,e**) for different initial conditions, for two values of 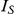 (vertical lines gray lines in **a**). The initial conditions are shown as empty dots near the left axis and the fixed points are shown on the right portion of each diagram.

Qualitatively different behaviours can be observed in the CB and DD models as a function of 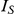. For instance, 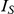 = 383 pA yields a bistable DD model (Fig. 3b), but its CB counterpart is monostable (Fig. 3d). In contrast, 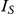 = 675 pA yields monostability in the form of repetitive spiking for the DD model (Fig. 3c), but bistability for CB (Fig. 3e). Both models display repetitive spiking for large enough values of 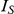 and both models block-depolarize at some point. However, these sustained oscillations emerge and stop through different mechanisms in the two models for a given 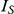.

### Ramp stimulation

**Figure 4:**
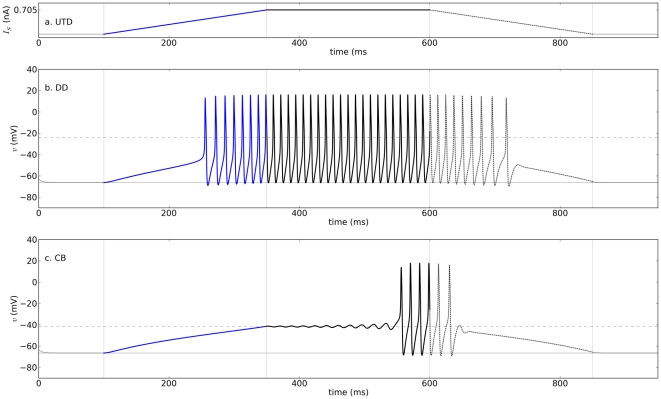
Dynamics of the model with DD currents and UTD stimulation. Panels **a–c** show, respectively, the current stimulus, membrane potentials of DD, and CB models as a function of time. Epochs last 250 milliseconds each (slope 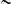
2.82 nA/ms), 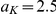, maximum stimulus amplitude 705 pA, and all other parameters as in Table 2.

Ramp stimulation (see UTD protocol and Fig. 4a) has the advantage of not causing the artificial one-dimensional 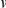-shift caused by square pulse stimulation, allowing the study of rest-to-spiking transitions while all the state variables of the system are changing. Importantly, the *recruitment current* in these conditions may be smaller than the 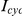 predicted for constant 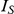 (this is called *slow passage through Hopf* [*62*], *Fig. 4b–c* *)*. Further, the mechanisms by which repetitive spiking starts when 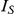 is a constant (i.e. square pulse stimulation) are different in comparison to those predicted for ramp currents. For illustration, consider a case where a *Top* stimulus slightly larger than the recruitment current in the CB model (Fig. 4b–c, 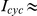
383 pA for DD and 608 pA for CB, *Top* amplitude = 705 pA). In this case, repetitive spiking starts after a relatively long delay in the CB model. The reason is that the ramp allows both variables of the system to change, thereby moving the system toward one of its attractors. At the start of the ramp, the system moves toward its nearest stable fixed point, which is a focus. Shortly before the *Top* amplitude is reached, the fixed point undergoes an AH bifurcation in which the focus becomes unstable, leaving a limit cycle as the only attractor of the system (see also Fig. S6). As a consequence, when the AH bifurcation occurs, the system starts oscillating away from the fixed point and toward the limit cycle. Note that repetitive spiking does not start through the FLC bifurcation as for constant 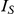 (compare to Fig. 3a,e) because the system stays close to the stable fixed point during the ramp. When the bistability regime starts (FLC), the system remains within the basin of attraction of the fixed point. Sometimes the current during the up-ramp becomes large enough to induce a depolarization block, but decreasing 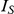 from there during *Down* may induce repetitive spiking. The amplitudes that result in block with a ramp current can also be different from the depolarization block stimulus predicted by the analysis in which 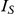 is constant.

**Figure 5:**
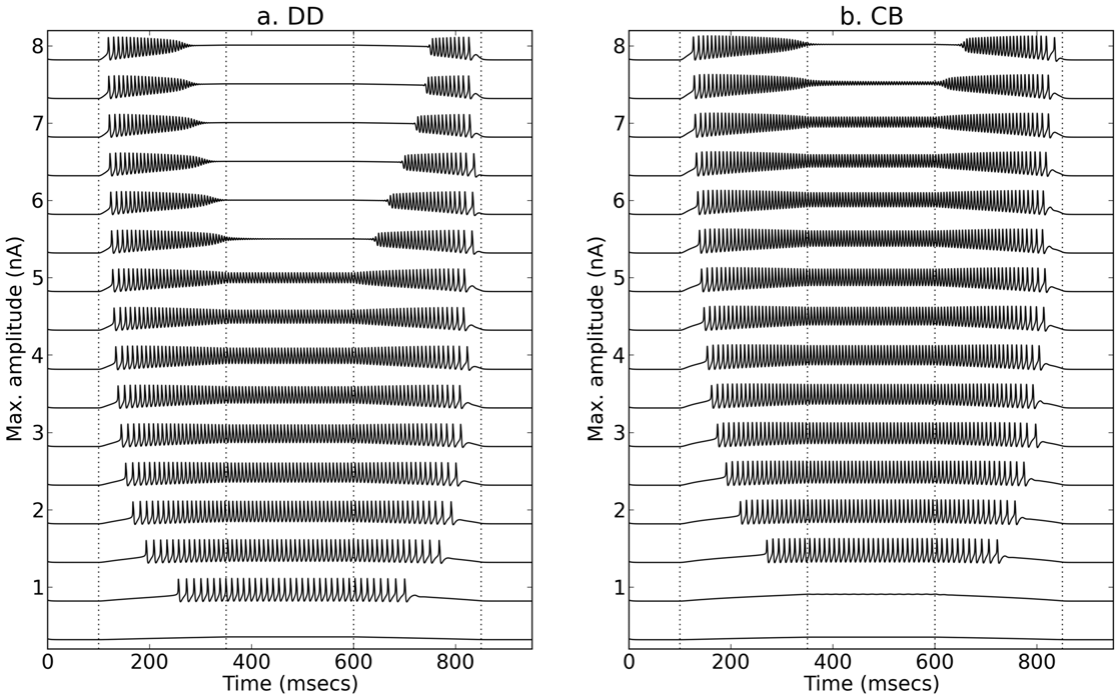
Response profiles of DD and CB models to UTD stimulation. Panels **a** and **b** show, respectively, the responses displayed by the DD and CB versions of the system Eqs. 5 and 6. All simulations with 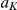 = 2.5 with epochs lasting 250 ms and maximum stimulus amplitudes between 0.2 and 8 nA in increments of 0.5 nA.

From comparing the *response profiles* to UTD stimulation with different maximal amplitudes and while keeping the same ramp durations (Fig. 5a–b), it is possible to generalize the observation that sustained spiking in the DD model starts for smaller maximal current amplitudes in comparison to the CB model. The two models show two kinds of *hysteresis*: (1) with respect to the recruitment and de-recruitment current amplitudes, and (2) with respect to the recruitment and de-recruitment firing rates. As a rule of thumb for slow ramps, the recruitment current is more likely to be smaller than the de-recruitment current with a larger recruitment firing rate larger in comparison to the de-recruitment rate (see Fig. 5 and also Fig. S6c,f); the trend reverses for steep ramps (compare traces with low and high *Top* amplitudes in Fig. 5a–b). Also, the current at which spiking ceases during *Up* can be different than the current for which spiking starts during *Down* (upper traces of Fig. 5a).

There is thus some qualitative agreement in the predictions of the behavior of the DD and CB models for constant 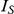 and ramp stimulation. As predicted for constant 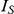, the recruitment current for the DD model is smaller relative to that of the CB model and within a smaller range (Fig. 4b–c and Fig. 5a–b).

### Different responses to the same synaptic input

The general differences in excitability described previously should also hold when 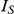 represents synaptic input. Two questions of particular importance for the study of motor neuron behavior, and for network models in general, are whether recruitment with excitatory synaptic input occurs for smaller synaptic drive in the DD model in comparison to the CB model.

**Figure 6:**
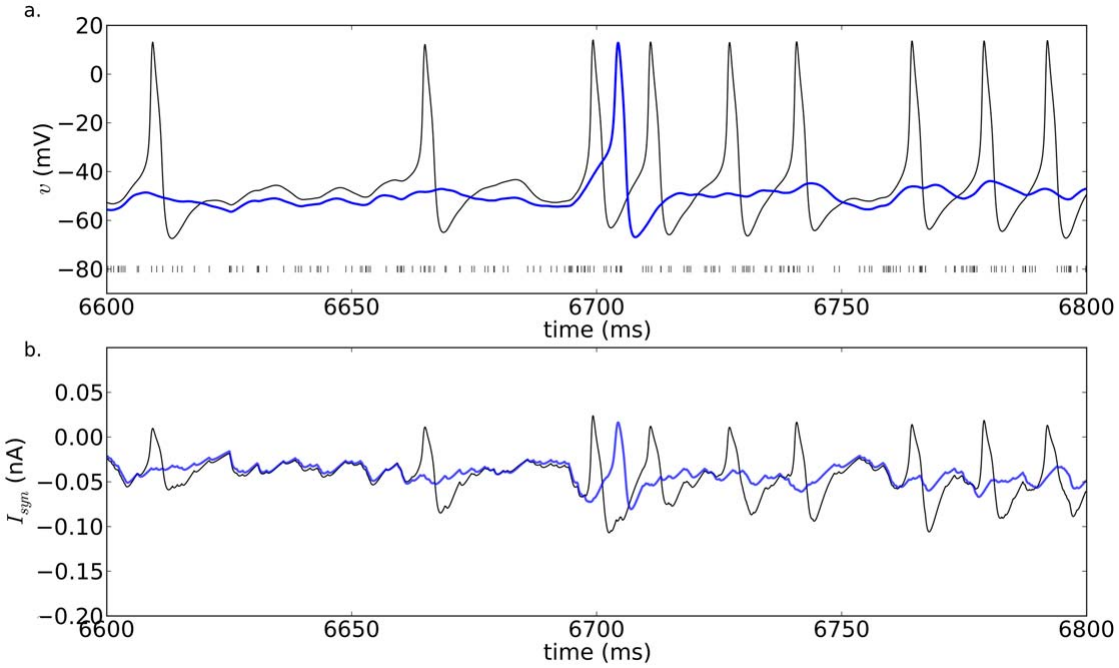
DD and CB action potentials in response to fast excitatory synaptic input. (**a**) Membrane potential for the DD (black) and CB (blue) models, and presynaptic spike times (vertical dashed lines). (**b**) Post-synaptic current. Parameters: 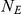 = 150, 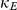 = 10.

To compare the response profiles of the two models, simulations were performed assuming 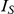 represents fast excitatory synaptic input ( Eqs. 7 and 8, and Table 1). The effects of synaptic drive on the two models are compared by increasing the number of synaptic contacts. The input axons, and hence their spiking activity, were assumed to be the same for the DD and CB models. On the postsynaptic end, the maximum amplitude, reversal potential, activation and inactivation constants, average number of active synapses, and the relative contributions of all channels, including the ones mediating synaptic input, are all identical in both models. The same excitatory synaptic input given to both models (Fig. 6) produces similar fluctuations in their membrane potential (Fig. 6a) with nearly identical synaptic currents except those around spike times (Fig. 6b). Nevertheless, in agreement with the earlier recruitment of the DD model shown in the previous analysis, the DD model in the example illustrated in Fig. 6 fires more action potentials than the CB model for the same number of input synapses.

**Figure 7:**
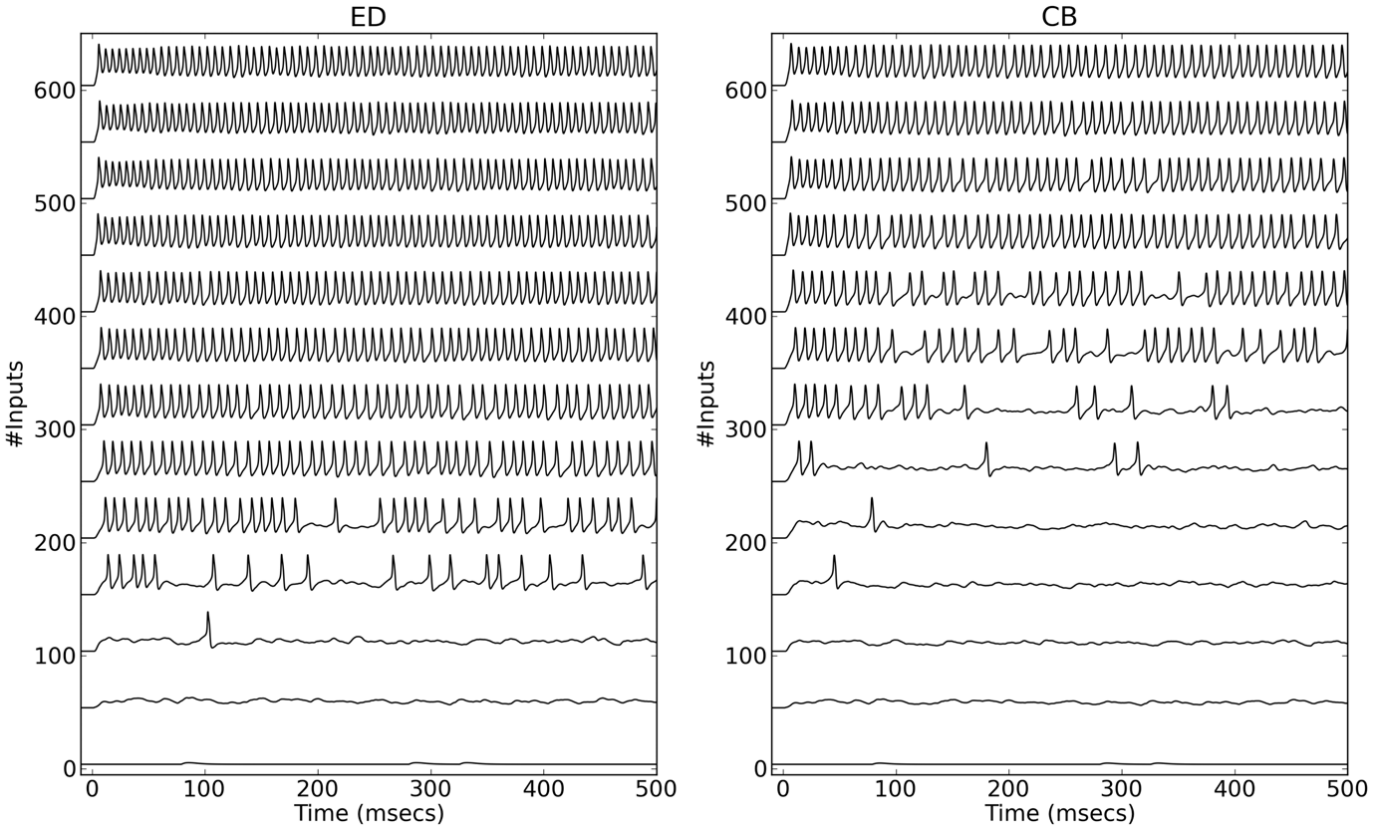
Profiles of DD and CB responses to excitatory synaptic input. All simulations with 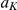 = 2.5 with increasing numbers of excitatory synapses assuming the average number of activated synapses per input axon 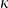 = 10.

To generate examples of the response profiles for the two models as a function of increasing excitatory input, the dynamics of the DD and CB membrane potentials were simulated for different numbers of excitatory axons (Fig. 7) assuming 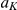 = 2.5. Regular spiking responses (with relatively constant inter-spike intervals) occur when approximately 250 inputs excite the DD model. In contrast, the CB model starts producing regular spiking with approximately 450 inputs. In other words, the smallest number of activated excitatory synapses necessary to trigger sustained spiking in the DD model under consideration is smaller (by a factor close to 2) than the number of synapses needed to elicit repetitive spiking in the CB neuron. Therefore, the *same synaptic input* produced very different responses in these two model neurons having the same populations of ion channels (Fig. 7).

The difference in the number of inputs required to recruit DD or CB neurons could have an important impact on the output properties and computations performed by small networks with a few thousand neurons.

## Discussion

### Advantages of the drift-diffusion model

The formulation of currents based on DD from Eq. 3 represents a theoretical improvement over CB models. One consequence of taking diffusion into account is that currents in the DD model display rectification and other properties that cannot be observed in CB formulations without increasing the dimensionality of the system. Therefore, in comparison to CB models, DD formulations give more realistic representations of excitable membranes that do not require a large increase in computation. DD models could thus advance our current understanding of dynamical behavior in single cells and networks. For instance, the formulations shown here can be used to infer patterns of synaptic connectivity from knowledge about the input-output properties in cells or specific information about correlation patterns in their activity as done in [35]. In addition, the normalizations used in this article allow estimations of relative patterns of channel expression.

As pointed out in the seminal work by Goldman [11], by researchers in cardiophysiology (see for instance see Na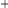, and K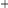 currents in [63]) and neurophysiology [14], [15], [64], the constant field approximation describes the voltage-dependence of currents better than the CB approach. Recall the Goldman constant field approximation is a particular case of the DD formulation used here. Evidence indicating that the DD approach is better in general can be found in several reports containing IV relationships with tails of hyperbolic sine shape. To mention a few instances, see recordings from photoreceptors [65], calcium channels [66], sodium channels [67] in *Drosophila*, snail neurons [68], in the mammalian cortex [69], and even in glial cell recordings as reported in [70]. The DD models have the advantage that measurements can be made directly from the currents recorded without extra calculations of maximal conductances. Such conductances are obtained as slopes of the current-voltage relationship, assuming current is the product of a conductance and a voltage difference (electrical drift only). When using the DD formulation, the maximal currents from voltage-clamp experiments can be directly fit with the model because the leading coefficients in the DD formulation are already in units of current and no extra calculations are needed. This “out of the box” behavior is reassuring because it enables the direct translation from recordings to computational models.

A complementary comparison between the DD and CB models should be done, however, against experimental measurements. One way in which it would be possible to decide whether to use DD or CB models would be to compare the response profiles of a cell membrane having blocked as many currents as possible, except the transient Na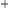 and delayed-rectifier K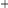 currents. Preparations like the squid giant axon might be ideal in this regard [14], [15]. An alternative approach could be to use an exogenous expression system to construct an excitable cell and test the two models there. The idea in general is that the basic input-output properties of the recordings should be fit with the two models along with spike shapes and firing rates. The channel kinetics should be determined from voltage-clamp experiments. To determine the relative contribution of the channels, 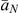 can be estimated from the maximum 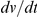 and 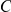. The relative contribution of the K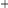 current 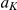 can then be found by matching the shape of the total steady state current. The resulting bifurcation profile (e.g. a fixed point curve like those from Fig. 2 a–b) would then be assigned to the recording. Once the bifurcation profile that matches the data has been determined, the stimulus currents giving repetitive spiking with both square pulses and with ramps should be better predicted by either the DD or CB model. In consideration of the more realistic representation of transmembrane current provided by the DD approach, the author hypothesizes that the DD model would yield more accurate predictions.

### Mathematical relationship between the DD and CB models

The whole-membrane behavior of the DD model shares many of the properties of CB models in that it contains parameters that can be found experimentally and displays dynamics observable in excitable cells. As shown in previous paragraphs, the two models are mathematically related: the CB formulation for current is a linear approximation of the DD formulation around the reversal potential for the current. In agreement with previous reports [14], [15], the divergence between the CB and DD currents near typical resting potentials can be further decreased when channel gating is taken into account. As a result, the DD and CB versions of the system Eqs. 5–6 display some qualitatively similar behaviors when observed macroscopically, but over different ranges of parameters. However, the two models with identical ion channel populations display qualitatively and quantitatively different transitions in their behaviors (i.e. the CB and DD models are *not* topologically equivalent).

### Similar but not same excitabilities with the same channel populations

The DD and CB models are compared by setting the relative contributions of the channels to be identical in both models. One of the most obvious differences between the DD and CB models is their range for repetitive oscillations. In general, if the ratio between the maximum K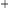 and Na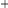 current amplitudes is larger than 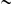
1.2, then the DD model responds with action potentials for smaller external input currents found within a smaller range compared with the CB model. The number of input axons that causes sustained spiking is smaller, and within a narrower range in the DD model in comparison to the CB model. The DD membrane is thus more excitable, and responds within a smaller input range, than the CB membrane. Further, if the excitability type is defined as the kind of rest-to-spiking transition observed while the external current increases smoothly (e.g. ramp), the DD and CB models represent membranes with the same populations of channels and different types of excitability (see for instance Figs. 3 and 4).

Interestingly, both models display two different hysteresis-related behaviors with ramping inputs. In very general terms, the recruitment current is larger than the de-recruitment current if the steepness of the ramps is shallow. In contrast, the recruitment current is smaller than the de-recruitment current when the ramps are very steep. For particular interest, the two models display what has been reported as a *slow passage through Hopf* [71] in which a ramp current triggers sustained oscillations for current amplitudes smaller than 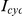, which can be predicted by bifurcation analysis using the stimulus current as the bifurcation parameter. A modified version of the slow passage through Hopf was also observed in simulations of excitatory synaptic input, as the total synaptic current that triggered spiking was also considerably smaller than the 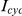 of the system.

#### An important remark related to firing rate hysteresis relevant for motor control

Note the only persistent current in the model is the delayed rectifier K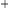 current. Therefore, the firing rate hysteresis and bistability regimes observed in the CB and DD models presented here are not the result of having persistent Na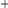 or Ca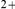 currents (see [72], [73]), thus highlighting the importance of explicitly distinguishing firing rate hysteresis and bistability behaviors from the presence of persistent inward currents in a membrane.

### Model specificity and extensions

The models are constructed based on *Drosophila* data. The choice of the specific channels and animal model is based on the large availability of whole-cell patch clamp recordings, which provide measurements that can be directly used as model parameters. In addition, the use of *Drosophila* data allows interpretations in terms of specific channel genes. Since the parameters used here are representative of central neurons in *Drosophila* the post-synaptic currents are assumed to be mediated by fast cholinergic receptors [44]. It should be noted, however, that the biophysical properties of homologous channels in vertebrates have also been characterized (for sodium channels see [74]; potassium channels [37], [40], [75], [76]; cholinergic neurotransmission [77]; central synapses mediated with glutamate receptors [32], [78], [79]). Therefore, the approach taken here can easily extended to represent other channels so the formulations of membrane potential can be adjusted to model other systems.

### Data fitting and modeling techniques

For theoretical interest, writing the model in terms of ratios using the normalizing amplitude 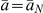 allows fixing one of the maximal current amplitudes in the model while fitting the maximum 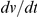 to data. This way, the whole-membrane behavior can be tuned in terms of the relative contributions of the different channels guided by bifurcation theory, thus providing an alternative to brute-force fitting algorithms and other statistical approaches [80]. Furthermore, the conceptual improvement in the formulation for single channels is extended to facilitate quantitative agreement at the whole-membrane level.

The analysis and fitting procedures presented here can guide studies geared toward understanding cellular responses recorded in the laboratory under genetic and pharmacological manipulations. In this respect, it is worth remarking that this is one of only a few modeling efforts that incorporates data from an identified neuron in a single model system in which both the ion channel genes and their biophysics are known. Importantly, the results shown here in regard to the differences between the DD and CB models also hold if the models are adjusted to match the cellular dynamics in other model organisms. For instance, if the parameters are adjusted so that the membrane has the input resistance and capacitance measured from a vertebrate cell of interest, with fast-transient Na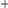 and K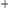 delayed rectifier channels (e.g. Nav1.2 and Kv2.1) and fast AMPA synapses, the DD and CB models will display the same general qualitative differences in excitability presented here. If the specific genes are not known, the phenotypical behavior of currents can still be associated with specific families of proteins grouped by function (say, mediating fast-transient Na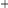 currents), which can be modeled with the approach shown in this article. Another extension of this work could be made to incorporate neuronal structure and address issues related to the targeting of channels to specific submembrane domains. One further extension would involve the construction of networks formed with different cell types having realistic synaptic interactions to study the role played by synaptic efficacy, number of input synapses, and other variables on the input-output properties of networks.

### Final remarks

The construction of the membrane models in this article rests on the hypothesis that the relative presence of channels in the membrane determines, to a large extent, what we could refer to as the *electrophysiological profile* of a cell [27], [81]–[83]. This theoretical principle was used here to compare and contrast responses to current injection and synaptic input of two membranes expressing identified ion channel genes with known biophysical properties. The non-topological equivalence between the DD and CB models predicts qualitatively different behaviors for the same patterns of channel expression. As shown here, the nonlinearities in the DD formulation for transmembrane currents can fundamentally change the spike-generating mechanisms and sensitivity to external stimulation in the whole DD model. Of particular importance, the two models generally display different spike-generating mechanisms as a function of the input current, synaptic or applied. As a consequence, the input and output firing rates of DD and CB cells within network models will be very different on any given architecture, potentially giving rise to very different results and interpretations. These differences are important because the intrinsic properties of neurons (and excitable cells in general) shape the activity of cellular networks to which they belong. Conversely, the network also influences the electrophysiological profile of single cells through the population of channels that mediate synaptic input and also through other modulatory influences. This is a subject that warrants a further and more careful examination currently underway.

The results presented here highlight the importance of exploring the different responses produced by two kinds of extensions of the DD models presented here: spatially detailed models and networks. These two extensions (and others) are likely to yield very different results and predictions in comparison to those from existing CB models, potentially prompting a reevaluation and possibly, a re-interpretation of accepted theories originated from network models of nervous function.

## Supporting Information

Figure S1
**Fitting of voltage clamp data from Shab channels** [**39**] **.** The data (black dots) were digitized from the original publication and fitting was done with a python script. The blue curves are fits to the data parameters 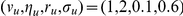. Middle, steady state activation from the tail currents shown in the top panel (black dots), and average from all recordings (white dots). The lower panel shows the time constant fit.(TIF)

Figure S2 **Cholinergic Synaptic input.**
(TIF)

Figure S3
**Steady state currents for the DD and CB models for** 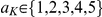 **.** The top curve in each panel of corresponds to 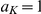, the bottom curve corresponds to 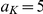, and the vertical gray line indicates the total current is zero.(TIF)

Figure S4
**Trajectories of membrane potential with different levels of depolarization from rest (** 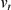 **).** DD (solid) and CB (dashed) for 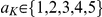 shown from left to right. The upper panels show the membrane potential and fixed points for the two models (DD solid lines and dots, CB dashed lines and circles). The lower panels show the corresponding currents (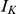, 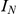, 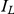, 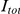 in red, blue, green, and black, respectively, and 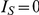). (**a**) 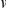 shifted 26 mV. (**b**) 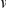 shifted 34 mV.(TIF)

Figure S5
**Profile of responses to square pulses of different amplitude.** The pulses lasted 200 milliseconds with 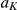 = 2. The minumum pulse amplitude was 0.025 nA, the steps where 0.5 nA.(TIF)

Figure S6
**Comparison of phase trajectories and instantaneous firing rate during UTD stimulation.** Panels **a** and **c** show, respectively, DD and CB trajectories in the phase plane 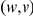. The solid gray curves in panels represent the 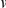 and 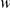-nullclines in the absence of stimulation. The dashed gray line represents the 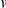-nullcline during *Top*. **b** and **d** Graphs of 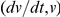 for DD and CB models, respectively. The horizontal line illustrates the 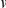-location of the fixed point. **c** and **f** Instantaneous firing rates as a function of time. The horizontal lines illustrate the recruitment and de-recruitment firing rates (blue and black, respectively).(TIF)

Text S1 Overview of the derivation of the expressions for current driven by electrodiffusive transport and notes about bifurcation analysis.
(TEX)
