# Supplementary figures and images for: Membranes with the Same Ion Channel Populations but Different Excitabilities
Source: PLoS One. 2012 Apr 16;7(4):e34636. doi: 10.1371/journal.pone.0034636 (PMC3327720; doi:10.1371/journal.pone.0034636)

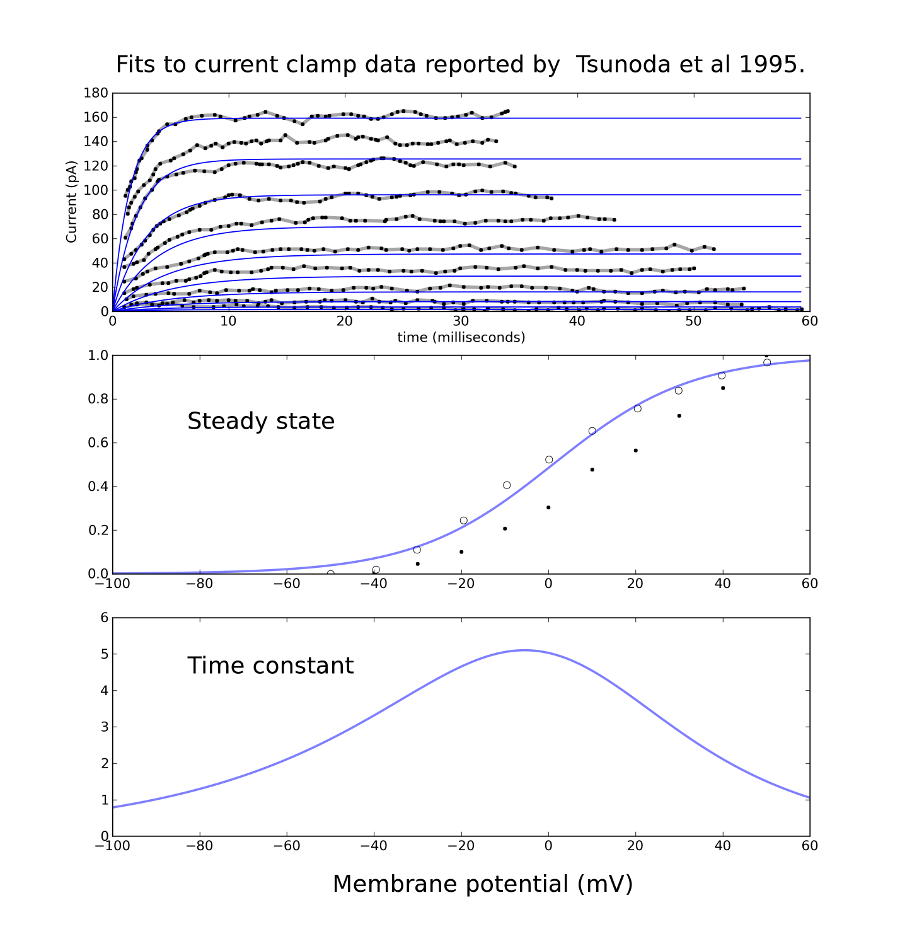

Supplement: Figure S1 — Fitting of voltage clamp data from Shab channels [39] . The data (black dots) were digitized from the original publication and fitting was done with a python script. The blue curves are fits to the data parameters . Middle, steady state activation from the tail currents shown in the top panel (black dots), and average from all recordings (white dots). The lower panel shows the time constant fit. (TIF) [file pone.0034636.s001.tif]

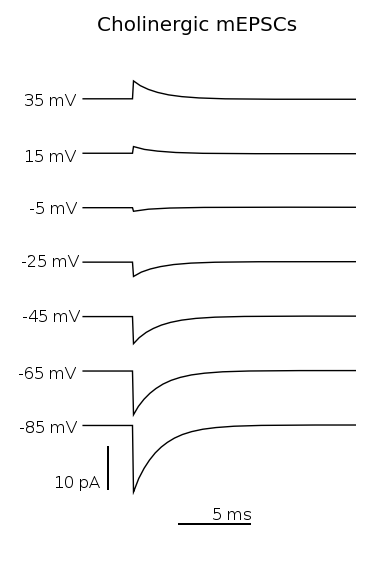

Supplement: Figure S2 — Cholinergic Synaptic input. (TIF) [file pone.0034636.s002.tif]

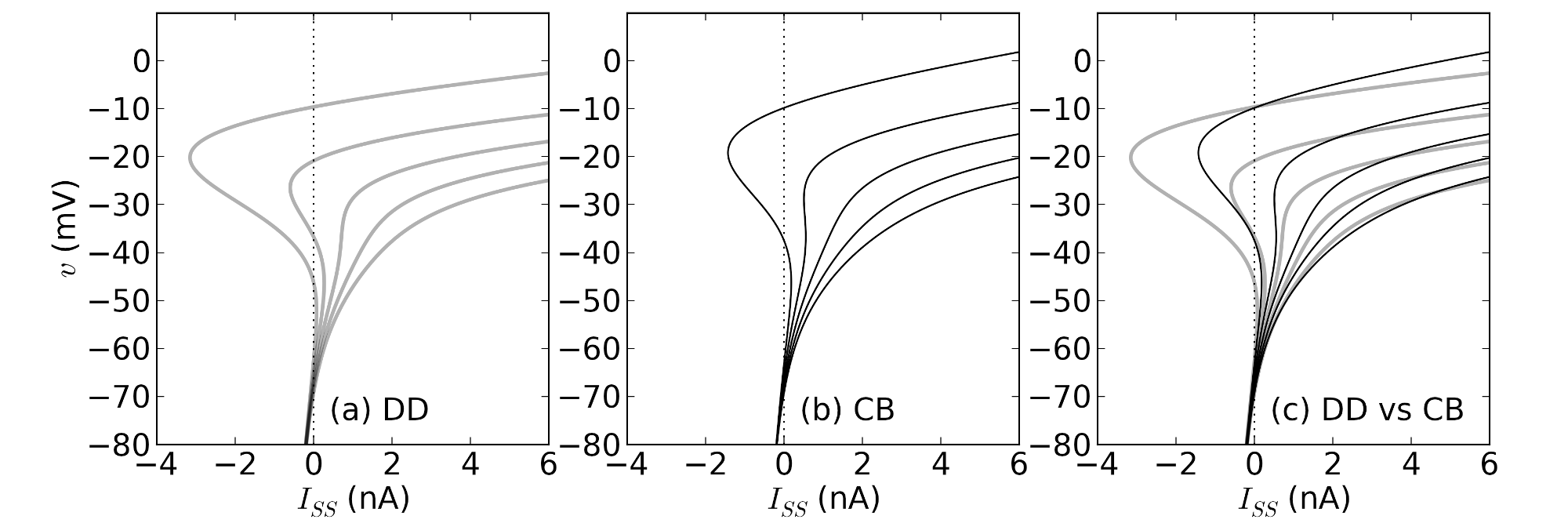

Supplement: Figure S3 — Steady state currents for the DD and CB models for . The top curve in each panel of corresponds to , the bottom curve corresponds to , and the vertical gray line indicates the total current is zero. (TIF) [file pone.0034636.s003.tif]

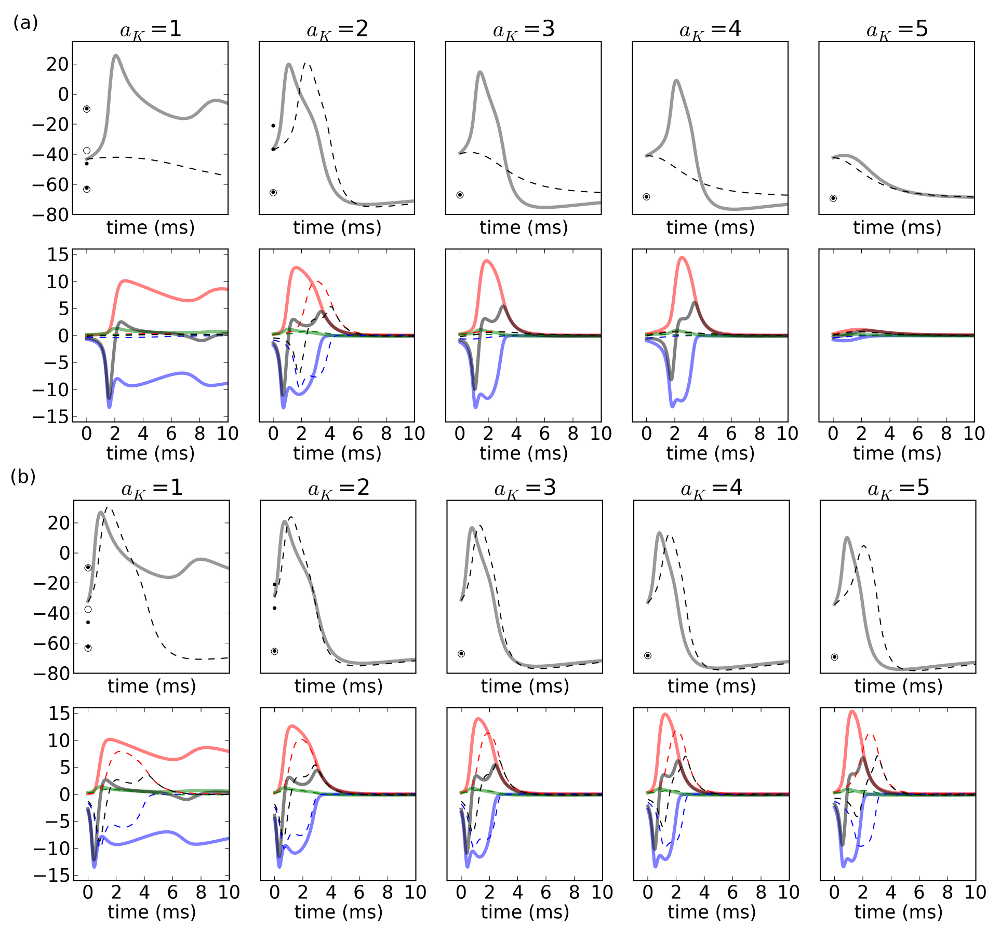

Supplement: Figure S4 — Trajectories of membrane potential with different levels of depolarization from rest ( ). DD (solid) and CB (dashed) for shown from left to right. The upper panels show the membrane potential and fixed points for the two models (DD solid lines and dots, CB dashed lines and circles). The lower panels show the corresponding currents (, , , in red, blue, green, and black, respectively, and ). (a) shifted 26 mV. (b) shifted 34 mV. (TIF) [file pone.0034636.s004.tif]

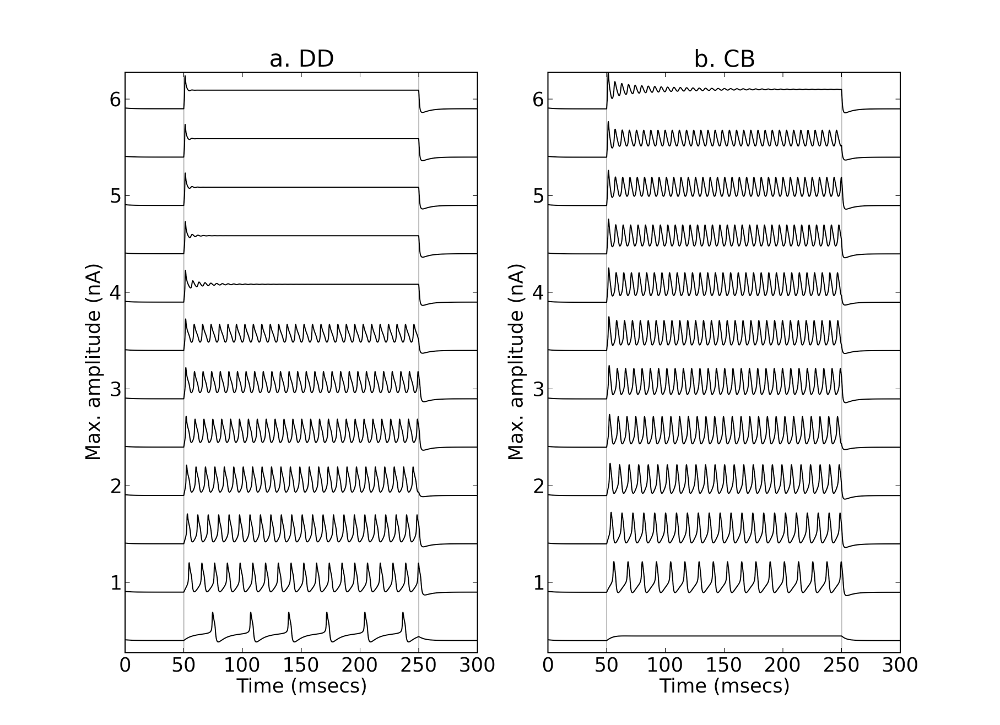

Supplement: Figure S5 — Profile of responses to square pulses of different amplitude. The pulses lasted 200 milliseconds with = 2. The minumum pulse amplitude was 0.025 nA, the steps where 0.5 nA. (TIF) [file pone.0034636.s005.tif]

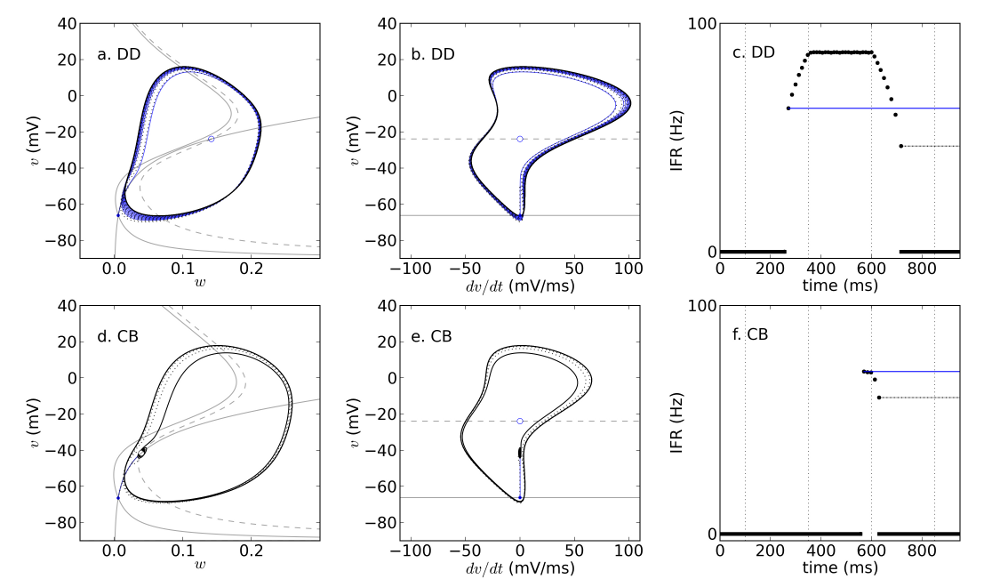

Supplement: Figure S6 — Comparison of phase trajectories and instantaneous firing rate during UTD stimulation. Panels a and c show, respectively, DD and CB trajectories in the phase plane . The solid gray curves in panels represent the and -nullclines in the absence of stimulation. The dashed gray line represents the -nullcline during Top. b and d Graphs of for DD and CB models, respectively. The horizontal line illustrates the -location of the fixed point. c and f Instantaneous firing rates as a function of time. The horizontal lines illustrate the recruitment and de-recruitment firing rates (blue and black, respectively). (TIF) [file pone.0034636.s006.tif]
